# Expanding Genetic and Clinical Spectra of Inherited Retinal Dystrophies: Identification of Three Novel *PRPH2* Variants

**DOI:** 10.3390/biomedicines13071531

**Published:** 2025-06-23

**Authors:** Raffaella Cascella, Jacopo Sebastiani, Claudia Strafella, Giulia Calvino, Sarah Andreucci, Michele D’ambrosio, Stefania Zampatti, Jung Hee Levialdi Ghiron, Benedetto Falsini, Andrea Cusumano, Emiliano Giardina

**Affiliations:** 1 Genomic Medicine Laboratory UILDM, IRCCS Santa Lucia Foundation, 00179 Rome, Italy; 2 Department of Chemical-Toxicological and Pharmacological Evaluation of Drugs, Catholic University Our Lady of Good Counsel, 1000 Tirana, Albania; 3 Macula & Genoma Foundation Italy, 00196 Rome, Italy; 4 Department of Science, Roma Tre University, 00146 Rome, Italy; 5 Macula & Genoma Foundation USA, New York, NY 10017, USA; 6 Ophthalmology Unit, Department of Experimental Medicine, University of Rome Tor Vergata, 00133 Rome, Italy; 7 Department of Biomedicine and Prevention, Tor Vergata University, 00133 Rome, Italy

**Keywords:** *PRPH2*, inherited retinal dystrophies, WES, OCT, ERG

## Abstract

**Background/Objectives**: Pathogenic variants in the *PRPH2* gene are implicated in a wide spectrum of Inherited Retinal Dystrophies (IRDs), which show significant phenotypic heterogeneity. This study combines genomic, clinical, and instrumental data, including BCVA, OCT, ERG, and visual field testing, using a multimodal approach to identify known and novel *PRPH2* variants, with the aim of refine genotype–phenotype correlations and improving the diagnosis of IRDs. **Methods**: A total of 830 Italian subjects diagnosed with IRDs by the multimodal clinical approach underwent WES on the Illumina^®^ Next-Seq 550 system. Genetic variants were evaluated by considering type, frequency, and pathogenicity using dedicated databases and bioinformatics tools. **Results**: WES analysis led to the identification of three novel *PRPH2* variants (c.653C>G, c.700T>C, c.121del) and seven previously reported variants (c.424C>T, c.458A>G, c.461_463del, c.493T>C, c.499G>A, c.612C>G, c.734dup) documented in public databases and the scientific literature. **Conclusions**: Our data confirm the wide spectrum of IRDs associated with *PRPH2* genetic variants and highlight the importance of integrating genetic, clinical, and instrumental data. This strategy enhances diagnostic accuracy and strengthens genotype–phenotype correlations, ultimately improving clinical decision-making and personalized patient management.

## 1. Introduction

Inherited Retinal Dystrophies (IRDs) are heterogeneous ocular disorders characterized by a progressive retinal degeneration. The prevalence of IRDs varies widely, with Retinitis Pigmentosa (RP) being the most common form, affecting approximately 1:4000 individuals worldwide [1]. Other dystrophies, such as Enhanced S-Cone Syndrome, are extremely rare with a prevalence of less than 1 in 1,000,000 [2]. IRDs are characterized by morpho-functional changes involving both the retina and the choroid, causing severe visual impairment. The most common symptoms include the appearance of dark spots in the visual field (scotomas), decreased visual acuity, night blindness, photophobia, abnormal colour perception, and visual field alterations [3]. IRDs differ in the age of onset, severity, rate of progression, and inheritance pattern among patients, reflecting the high heterogeneity of these disorders. IRDs display autosomal recessive, autosomal dominant, X-linked, mitochondrial, and digenic patterns of inheritance. Moreover, incomplete penetrance and variable expressivity further complicate clinical diagnosis and genotype–phenotype correlations [4]. More than 270 genes are known to be involved in the onset and progression of IRDs. In particular, these causative genes encode proteins responsible for various retinal cell structures, visual cycle regulation pathways, phototransduction processes, and photoreceptor maintenance [5]. In this regard, several IRDs have been associated with likely pathogenic/pathogenic variants in the *PRPH2* (peripherin2) gene, accounting for 7–23% of cases with an autosomal dominant inheritance. More than 200 *PRPH2* variants have been reported to be involved in the onset of RP, macular dystrophies, or central areolar choroidal dystrophy, displaying extensive phenotypic heterogeneity. To support this, genetic variants in the *PRPH2* gene are associated with mild, late-onset macular dystrophies, whereas others are linked to severe, early-onset retinal dystrophies [6]. The *PRPH2* gene is located on the short arm of chromosome 6 (6p21.2) and encodes a transmembrane glycoprotein called Prph2, crucial for the morphogenesis, maintenance, and stabilization of the Outer Segment (OS) disc rims in rod and cone photoreceptors [7]. The complete absence of Prph2 results in a failure of OS formation in rods, whereas partial inactivation leads to shorter and structurally disorganized OSs in both rods and cones [8,9]. This protein contains four transmembrane domains and an intracellular domain that creates intramolecular disulphide bonds mediating the assembly of Prph2/Retinal Outer Segment Membrane protein 1 (Prph2/Rom1) tetramers [10,11]. The assembly of the Prph2/Rom1 complex is crucial for the functional activity of the protein [12].

Considering the clinical and genetic heterogeneity of *PRPH2*-related diseases, genetic testing represents a valuable tool for supporting the clinical and instrumental diagnosis of IRDs. Given these premises, the study aims to identify disease-causing variants in the *PRPH2* gene and accurately link these genetic variations to specific disease manifestations in a cohort of IRD patients. The goal is to establish a precise genotype–phenotype correlation to enhance the understanding of *PRPH2*-associated retinal diseases, thereby improving the accuracy of diagnosis and prognosis. This would enable the development of a multimodal tool designed to support the clinical assessment of patients by combining *PRPH2* causative variants with clinical and instrumental data. Clinical data include patient history, reported symptoms, physical examination findings, and disease progression, while instrumental data encompass ophthalmic assessments such as BCVA (Best Corrected Visual Acuity), HR-OCT (High Resolution Optical Coherence Tomography), ERG (Electroretinography), and visual field testing. This comprehensive approach aims to enhance phenotype characterization and encourage the development of personalized therapeutic strategies tailored to individual patients.

## 2. Materials and Methods

### 2.1. Study Cohort and Ocular Examinations

This research study involved 830 Italian subjects with a clinical–instrumental diagnosis of IRDs recruited between the end of 2017 and 2022. The molecular test was performed at the Genomic Medicine Laboratory UILDM at the Santa Lucia Foundation IRCCS. The study was carried out according to the Declaration of Helsinki and written informed consent for both clinical and genetic testing was obtained from all patients. The study was approved by the Ethical Committee of Santa Lucia Foundation IRCCS (Approval No. CE/PROG.650). The cohort consists of 440 females and 390 males with an average age of 55.3 ± 14.8. Patients were examined with a multimodal clinical approach and they were subjected to a comprehensive ophthalmological examination, including BCVA, HR-OCT, ERG, CV FDT MD (Frequency Doubling Technology Visual Field Testing), and colour fundus photography, in order to define a detailed clinical diagnosis.

BCVA results were recorded both in LogMAR (Logarithm of the Minimum Angle of Resolution) and letter score formats. LogMAR provides a logarithmic representation of visual acuity, where lower values indicate better visual performance. A LogMAR value of 0.0 corresponds to 10/10 visual acuity (decimal scale), whereas higher values represent progressively worse visual acuity. The letter score indicates the number of letters the patient can read correctly on a standard chart, with a maximum score of 85 letters corresponding to a LogMAR of 0.0. Moreover, OCT is a non-invasive imaging technique that utilizes light waves to produce high-resolution cross-sectional images of the retina and the optic nerve. OCT provides quantitative measurements of retinal thickness, total retinal volume, optic cup depth, and retinal nerve fibre layer distribution. ERG is essential for assessing retinal function, differentiating between rod and cone photoreceptor activity. Retinal function is assessed by recording electrical responses to light stimuli. These responses are categorized into scotopic ERG (evaluating rod function) and photopic ERG (assessing cone function). Key parameters include amplitude (µV), which reflects the strength of the retinal response to the light stimulus, and peak time (ms), which represents the speed of retinal processing after light stimuli. CV FDT MD assesses global visual function by calculating the average deviation of a patient’s visual field from age-matched normal values. A value equal to or close to zero indicates normal sensitivity; negative values express reduced visual field sensitivity; positive values are rare and may suggest either higher sensitivity or potential measurement errors. According to instrumental evaluation parameters, patients were diagnosed with macular dystrophy, cone dystrophy, cone–rod dystrophy, and RP. In parallel with the ophthalmological examination, genetic counselling was provided to all patients for collecting personal and family history data.

### 2.2. Whole Exome Sequencing (WES) and Bioinformatics Analysis

Genomic DNA was isolated from buccal swab [13] using the MagPurix Automatic Extraction System (Resnova, Rome, Italy) according to the manufacturer’s instructions. DNA concentration and quality were assessed by DeNovix spectrophotometer (Resnova). The Illumina NextSeq 550 system (Illumina, San Diego, CA, USA) was utilized to perform the WES analysis. In particular, 30–50 ng/μL of DNA was employed for library preparation using Illumina^®^ DNA Prep with Enrichment and Tagmentation kit according to the manufacturer’s instructions. The obtained libraries were sequenced at 2 × 100 bp and the sequencing quality of the resulting data was expected to reach a quality score >30 (Q30) for ~80% of total called bases. The Integrative Genomics Viewer (v.2.7.2) was employed for the quality and coverage evaluation of the identified variants. The BaseSpace Variant Interpreter (Illumina, v. 2.15.0.110) was utilized for variant annotation. WES data were analyzed, focusing attention on 85 genes known to be implicated in IRDs (Supplementary Table S1). The variants with a minimum coverage of 20X were included in the subsequent analysis. Genetic variants were classified according to type (nonsense, missense, frameshift, splicing) and frequency. The Minor Allele Frequency (MAF) of the variant is <0.001, according to data from publicly available databases (gnomAD). Further evaluation of the variants involved assessing their position within regulatory regions or protein domains, as well as their predicted pathogenicity using different bioinformatics tools (Uniprot and Decipher). The variant classification was performed according to the ACMG (American College of Medical Genetics and Genomics) Standards and Guidelines and ACGS (Association for Clinical Genomic Science) Best Practice Guidelines, which discriminate among benign, likely benign, Variant with Uncertain Significance (VUS), likely pathogenic, and pathogenic [14,15]. Variants identified in genes other than *PRPH2* were classified as benign or likely benign according to ACMG/ACGS criteria and were therefore excluded from further analysis. Clinical interpretation was supported by several databases, including Varsome, Clinvar, ClinVar Miner, InterVar, LOVD, Decipher, and Franklin. For novel missense variants, functional predictions were evaluated by REVEL (Rare Exome Variant Ensemble Learner), which is a metapredictor tool able to integrate different predictive scores (MutPred, FATHMM v2.3, VEST 3.0, PolyPhen-2, SIFT, PROVEAN, MutationAssessor, MutationTaster, LRT, GERP++, SiPhy, phyloP, phastCons) [16]. Furthermore, the impact of novel missense variants on protein function and structure was evaluated by Varsite [17], Missense3D [18], HOPE [19], and DUET [20]. Variants with a clinical significance were confirmed by direct sequencing (Supplementary Figure S1) using BigDye Terminator v3.1, BigDyeX Terminator, and ABI3130xl (Thermofisher, Waltham, MA USA) according to the manufacturer’s instructions. In addition, segregation analysis was performed for all the available family members in order to evaluate variant inheritance patterns.

## 3. Results

**Figure 1:**
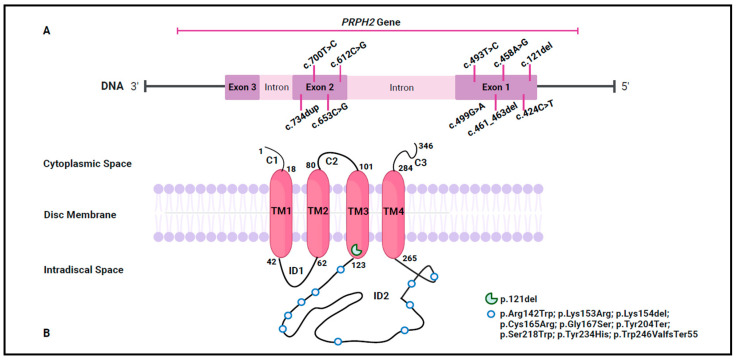
An illustration of the identified variant. (**A**) A schematic representation of the genetic variants located on the *PRPH2* gene. (**B**) A schematic representation of the genetic variants located on the Prph2 protein. The Prph2 peptide chain comprises extradiscal (C1, C2 and C3), transmembrane (TM1, TM2, TM3, and TM4), and intradiscal (ID1 and ID2) space regions. The numbering denotes the amino acid positions at the boundaries of the protein domains and the identified variants are marked by a green incomplete circle and light blue circles. This figure was created with Biorender.com (accessed on 2 January 2025).

Out of 830 tested patients, the analysis of WES data led to the identification of 10 *PRPH2* variants in 11 patients (1.3%). Specifically, three were novel (c.653C>G, c.700T>C, and c.121del), while the remaining seven (c.424C>T, c.458A>G, c.461_463del, c.493T>C, c.499G>A, c.612C>G, and c.734dup) had been previously reported in public databases (ClinVar, LOVD, and Decipher) and in the scientific literature. A detailed representation of the identified variants was reported in Figure 1A,B, highlighting their localization on both the gene and the corresponding protein.

Among the identified variants, six were missense (c.653C>G, c.700T>C, c.424C>T, c.458A>G, c.493T>C, and c.499G>A), two frameshift (c.734dup and c.461_463del), and two nonsense (c.121del and c.612C>G). These variants were investigated considering their frequency, bioinformatics prediction, protein structural impact, and ACMG/ACGS classification (Table 1).

The missense variant c.653C>G (p.Ser218Trp), located on the exon 2 of *PRPH2*, was found at the heterozygous state in a single patient (pt-1A) diagnosed with macular dystrophy, with ERG showing a reduced photopic response suggestive of cone dysfunction (Supplementary Table S2).

This variant was not reported in the main public archives such as ClinVar, ClinVar Miner, LOVD, Decipher, and Uniprot. According to the ACMG/ACGS, c.653C>G was classified as a pathogenic variant following these criteria: PM1 (located in a hot spot and/or critical and well-established functional domain), PM5 (novel missense change at an amino acid residue where a different missense change determined to be pathogenic), PP3 (multiple lines of computational evidence indicating a deleterious effect on the gene or its product), and PM2 (absent in Exome Sequencing Project, 1000 Genomes Project, Exome Aggregation Consortium). According to in silico predictions, this variant is likely deleterious and may impair normal protein function. Unfortunately, segregation analysis could not be performed due to the unavailability of additional family members.

The variant c.700T>C (p.Tyr234His) was localized in the exon 2 of *PRPH2* and was identified at the heterozygous state in a single patient (pt-2A) with cone–rod dystrophy. Instrumental data were collected at multiple time points. Longitudinal OCT evaluations revealed progressive retinal thinning. Moreover, BCVA of the right eye showed a gradual reduction in visual acuity, while the left eye showed a more pronounced impairment. ERG highlighted a progressive decline in both scotopic and photopic responses (Supplementary Table S2). The c.700T>C variant was annotated in ClinVar and Decipher and was not described in ClinVar Miner, LOVD, or Uniprot. Following the ACMG/ACGS criteria, the c.700T>C variant was described as VUS based on applying PM1, PM2, and PP3 criteria. Although some prediction tools indicated a detrimental effect for this variant, analysis using Missense3D and Varsite suggested that this amino acid substitution may not affect protein function. During genetic counselling, the proband referred a maternal uncle with a diagnosis of macular dystrophy; however, DNA samples were not available to perform segregation analysis.

**Figure 2:**
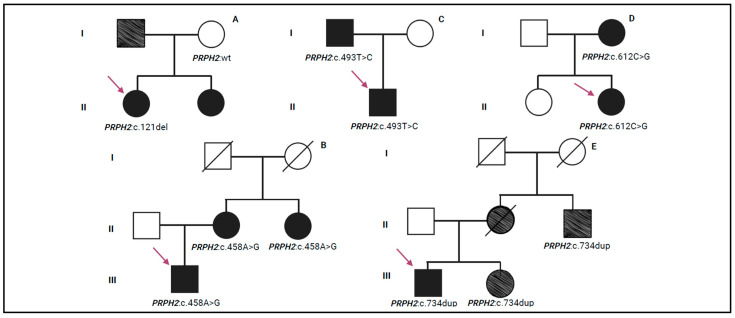
Pedigree trees illustrating the autosomal dominant transmission of *PRPH2* variants within the families. The families labelled (**A**–**E**) are those for which segregation analysis was conducted. The purple arrow indicates the proband. The partially coloured symbol indicates a condition of low vision reported by the proband during genetic counselling. Roman numerals in the pedigree indicate the generations. This figure was created with Biorender.com (accessed on 2 January 2025).

Concerning the variant c.121del (p.Leu41Ter), it was located in the exon 1 of *PRPH2* and was found at the heterozygous state in a single patient (pt-3A) with a diagnosis of retinal dystrophy. Unfortunately, no instrumental data are available for this patient. This variant was annotated in ClinVar, ClinVar Miner, and Decipher and was missing in LOVD and Uniprot. c.121del was classified as pathogenic according to the following ACMG/ACGS criteria: PVS1 (null variant with Loss Of Function, LOF, is a known mechanism of disease), PP5 (reputable source reports variant as pathogenic), and PM2. c.121del is a nonsense variant that creates a premature stop codon, resulting in an absent or disrupted protein product. During genetic counselling, the proband explained that her father experienced loss of vision and her sister received a clinical diagnosis of retinal dystrophy. Segregation analysis was performed only on the unaffected mother of the proband who was negative to the variant (Figure 2A).

Concerning the other missense variants (c.424C>T, c.458A>G, c.493T>C, and c.499G>A), they were identified in single patients at the heterozygous state and were localized in the exon 1 of the *PRPH2* gene. These variants were listed in ClinVar, ClinVar Miner, Decipher, LOVD, and Uniprot, except for c.493T>C that was not reported in Uniprot. Following the ACMG/ACGS criteria, these variants have been described as pathogenic. In particular, the criteria PP5, PM1, PM5, PP3, and PM2 were used to classify c.424C>T (p.Arg142Trp), identified in a single patient (pt-4A) with a diagnosis of retinal dystrophy. c.458A>G (p.Lys153Arg) was classified using the ACMG/ACGS criteria, PP5, PS3 (well-established in vitro/in vivo functional studies supportive of a damaging effect), PM1, PM5, PP3, and PM2, and was detected in a single patient (pt-5A) with RP. The segregation analysis identified the same variant in the patient’s mother (pt-5B) and maternal aunt (pt-5C) who were also affected by RP (Figure 2B). Unfortunately, instrumental data for pt-4A, pt-5A, pt-5B, and pt-5C patients were not available. Concerning the missense variant c.493T>C (p.Cys165Arg), it was identified in a single patient (pt-6A) with a positive family history of Stargardt-like macular dystrophy. Ocular examinations revealed a gradual decrease in retinal thickness between 2021 and 2022. Visual acuity remained good in both eyes throughout the observation period. Scotopic retinal function appeared relatively stable, while photopic ERG showed a slight improvement. However, visual field values demonstrated a clear progressive loss of sensitivity (Supplementary Table S2). c.493T>C was classified using the ACMG/ACGS criteria, PP5, PM5, PP3, PM1, and PM2, and it was reported as a disease-causing variant in different subjects with IRDs [21,22]. During genetic counselling, the proband reported that his father (pt-6B) received a clinical diagnosis of macular dystrophy. Segregation analysis was performed, confirming the presence of the c.493T>C variant in the father (Figure 2C). Unfortunately, ocular evaluations for this subject were not available. Regarding the c.499G>A (p.Gly167Ser) variant, it was identified in a single patient (pt-7A) with clinical signs consistent with rod–cone dystrophy; however, instrumental data were not available. The ACMG/ACGS criteria used to classify the variant were PP5, PM5, PP3, PM1, and PM2, supporting its classification as deleterious and likely to disrupt protein function. Despite the patient’s reported positive family history of visual impairment, a segregation analysis could not be performed. Concerning the nonsense c.612C>G (p.Tyr204Ter) variant, it was identified in a single patient (pt-8A) with a diagnosis of cone–rod dystrophy. Ophthalmic examination revealed reduced visual acuity, with a more pronounced decline in the left eye. Additionally, both scotopic and photopic responses showed reduced amplitude, while response times remained preserved. Furthermore, the visual field data confirmed widespread retinal damage, with severe peripheral sensitivity loss observed in both eyes (Supplementary Table S2). This variant was localized in the exon 2 of the *PRPH2* gene and the amino acid change created a premature stop codon, leading to the loss of protein function. It was reported in ClinVar, ClinVar Miner, Decipher, and LOVD. This variant was classified as pathogenic according to PVS1, PP5, and PM2 ACMG/ACGS criteria. Segregation analysis was performed on the proband’s family members, revealing the presence of this pathogenic variant in one of her daughters (pt-8B) (Figure 2D). The pt-8B patient showed normal OCT results in the right eye and a value above the normal range in the left eye. In addition, BCVA was also good with 82 letters in the right eye and 75 letters in the left eye. The ERG evaluation of the right eye showed a reduced amplitude and a slight delay in the peak time of the scotopic ERG. The photopic ERG also displayed a diminished amplitude, but the peak time remained normal. In contrast, the left eye’s ERG results were indicative of progressive retinal dystrophy, with a moderate reduction in scotopic amplitude and a significant delay in peak time. The photopic ERG for the left eye also showed reduced amplitude, although the peak time was normal. Additionally, visual field analysis revealed mild retinal sensitivity loss in the right eye and a slightly less pronounced impairment in the left eye. A summary of all ophthalmic parameters is provided in Supplementary Table S2.

Furthermore, WES identified c.461_463del and c.734dup variants in three different patients. c.461_463del (p.Lys154del) was found in a single patient (pt-9A) with a diagnosis of RP. Ocular evaluations were performed at different time points, including from 2017 to 2021. The retinal thickness values for the right eye fluctuated between 251 and 308 µm, while OCT measurements ranged from 263 to 293 µm for the left eye. Unfortunately, BCVA and ERG data were not available. Visual field testing in both eyes showed a considerable reduction in retinal sensitivity (Supplementary Table S2). The variant c.461_463del was located in the exon 1 of the *PRPH2* gene and was reported in ClinVar, LOVD, and Decipher. The variant was classified as pathogenic using the following ACMG/ACGS criteria: PP5, PM1, PM4 (protein length changes as a result of in-frame deletions in a non-repeat region), and PM2. In silico analysis predicted the c.461_463del variant to be disease-causing and likely to impair protein function. Unfortunately, segregation analysis was not performed. The variant c.734dup (p.Trp246ValfsTer55) was identified in two unrelated patients (pt-10A and pt-11A) with a diagnosis of cone–rod dystrophy and macular dystrophy, respectively. The instrumental data collected for the pt-10A patient revealed a preserved visual acuity. Visual field analysis revealed a decrease in visual sensitivity in the central retinal region of the right eye, with a milder reduction in the left eye (Supplementary Table S2). OCT and ERG data were not available. The ophthalmic evaluations of the pt-11A patient revealed a slight decrease in retinal thickness in both right and left eyes. BCVA measurements indicated preserved visual acuity (Supplementary Table S2). c.734dup was located in exon 2 of the *PRPH2* gene and it was not recorded in ClinVar or Decipher. This variant was classified as pathogenic according to ACMG/ACGS criteria, by applying PVS1, PP5, and PM2. The c.734dup variant was predicted to be disease-causing by in silico analysis, as it leads to the production of a truncated protein that may trigger nonsense-mediated mRNA decay (NMD). Furthermore, this variant may affect the function of the protein as reported in LOVD. Segregation analysis was performed exclusively in the family of patient pt-10A, identifying the c.734dup variant in both the sister (pt-10B) and the maternal uncle (pt-10C). In particular, during genetic counselling, the maternal uncle (pt-10C) was reported to have low vision (Figure 2E).

## 4. Discussion

Prph2 is a structural protein essential for the proper development of rod and cone OSs and thus for visual acuity. Prph2 is involved in rod and cone morphogenesis by promoting membrane curvature, flattening, and fusion. Moreover, Prph2 interacts with Rom1 to induce the development of OS rim domains. As is consistent with its crucial role in OS production, the aberrant expression of Prph2 has been associated with a wide spectrum of IRDs, which may show a high degree of variability in terms of age of onset and clinical signs, even in patients carrying the same likely pathogenic/pathogenic variants. This scenario complicates the precise diagnosis, the phenotype–genotype correlation, and, consequently, the prognosis in IRDs patients. In this regard, genetic testing can provide a valuable support to the clinical and instrumental diagnosis of IRDs. To date, the application of WES proved to be effective for identifying causative genetic variants and establishing specific correlations with the clinical phenotype of several genetic disorders, including IRDs.

On this subject, the present study exploited WES to investigate the occurrence of detrimental variants in *PRPH2* in a cohort of 830 Italian patients. As a result, the study allowed the identification of 10 disease-causing variants that were implicated in the onset and progression of *PRPH2*-associated diseases. These variants were employed to perform genotype–phenotype correlations to support and refine clinical diagnosis and confirm the presence of an autosomal dominant inheritance pattern. Among these, three variants (c.653C>G, c.700T>C, and c.121del) were novel and were not previously reported in the scientific literature. In particular, the missense variant c.653C>G (p.Ser218Trp) may affect the stability and folding of the Prph2 protein. In this regard, Serine (Ser) and Tryptophan (Trp) differ significantly in their biochemical proprieties. Ser is small, polar, and hydrophilic residue often involved in hydrogen bonding, contributing to protein stability. In contrast, Trp is a large, aromatic, and hydrophobic amino acid whose side chain may disrupt hydrogen bonding and interfere with the tertiary structure and overall protein conformation [19]. This conformational change is supported by DUET, which predicts alterations in protein stability (ΔΔG) following the substitution. In fact, this change tends to destabilize the protein by increasing the free energy (ΔΔG = −0.984 kcal/mol), indicating that the variant protein is less stable than the wild-type and that this reduced stability may significantly affect protein function. Overall, the data support the occurrence of protein misfolding, which may impair Prph2’s ability to maintain retinal membrane integrity and alter its interactions with photoreceptor membranes. Moreover, the Prph2 disruption may compromise the OS structure contributing to photoreceptor degeneration. As a result, the variant c.653C>G could impair the proper functioning of the retina, highlighting a possible progressive damage of the photoreceptor activity, primarily affecting the cones. This is consistent with its pathogenic classification and with the instrumental findings observed in the carrier patient, who exhibited a reduced response to high-intensity light stimulation. In particular, the reduction in ERG amplitude over time may trigger early retinal damage, highlighting the importance of ongoing monitoring to track the progression of this condition. The available data support the clinical diagnosis of macular dystrophy and the genotype–phenotype correlation suggests that the c.653C>G pathogenic variant is critically involved in cone dysfunction, thereby promoting retinal degeneration. A summary of the main ophthalmic and genetic findings is provided in Table 2.

**Figure 3:**
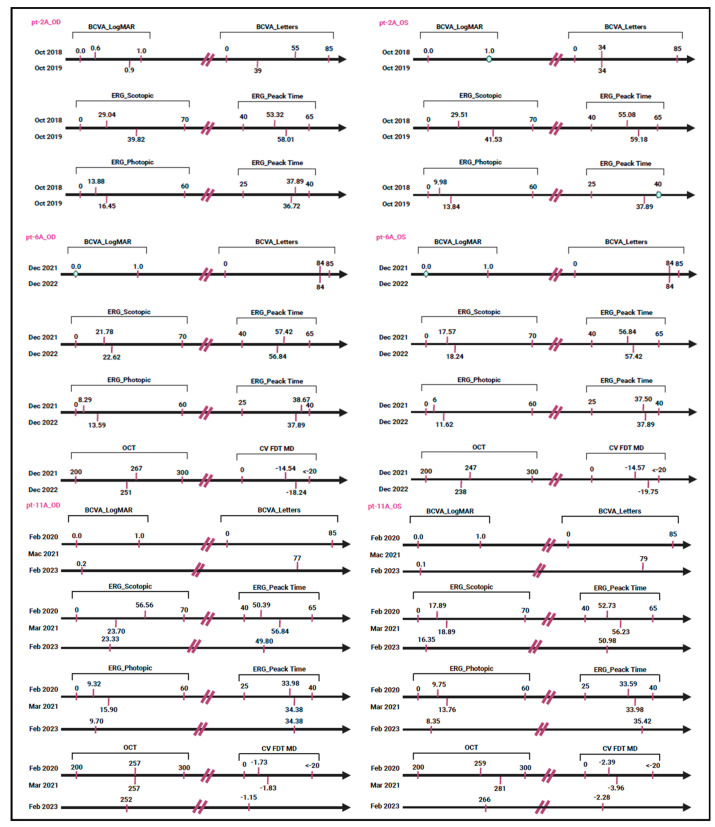
A longitudinal timeline chart depicting clinical progression in individuals evaluated at multiple time points. The circle indicates that the measurements remained unchanged over time. This figure was created with Biorender.com (accessed on 2 January 2025).

Concerning the missense variant c.700T>C (p.Tyr234His), its effect on the structure and function of Prph2 is debated. Available in silico analyses of protein stability have produced conflicting results, suggesting that the variant may have a minimal effect on the Prph2 structure [20]. From a biochemical perspective, Tyrosine (Tyr) is an aromatic, non-polar amino acid important for hydrophobic interactions in the protein core, whereas Histidine (His) is polar and positively charged, promoting electrostatic interactions. The substitution could therefore disrupt these connections, potentially altering the protein stability and function [19]. In particular, the variant may influence the protein’s ability to interact with membrane lipids in photoreceptor cells, a mechanism that remains to be experimentally confirmed. According to ACMG/ACGS criteria, the variant c.700T>C was classified as a VUS. While its clinical relevance remains unclear, some indications suggest a possible pathogenic role. The patient was clinically diagnosed with cone–rod dystrophy, characterized by progressive retinal tissue thinning and a gradual decline in visual function, suggesting a degenerative process. In particular, both cone and rod dysfunctions were documented, with a more severe involvement of the left eye (Figure 3).

These findings may indicate a genotype–phenotype correlation consistent with *PRPH2*-related disease (Table 2). Furthermore, the presence of a positive family history for visual impairment suggests a potential autosomal dominant inheritance, which is in line with known *PRPH2*-associated phenotypes. Given these data, PP4 criteria (patient’s phenotype or family history is highly specific for a disease with a single genetic etiology) may be carefully applied and the c.700T>C variant may be considered potentially disease-causing. However, it is clear that additional studies are needed to strengthen the interpretation and confirm its clinical significance.

Regarding the variant c.121del (p.Leu41Ter), it produces a premature termination codon, leading to NMD. This surveillance mechanism degrades premature mRNA transcripts that could hamper the formation of the Prph2 protein. Consequently, the presence of c.121del prevents the proper formation of Prph2, leading to retinal dysfunction and contributing to visual impairment. Furthermore, the early termination resulting in a non-functional protein may suggest a more severe phenotype and rapid retinal degeneration. This variant was identified in a patient with retinal dystrophy and was classified as pathogenic. However, the lack of available instrumental data made establishing a precise genotype–phenotype correlation difficult.

Furthermore, WES analysis revealed the presence of well-known pathogenic variants involved in *PRPH2*-related disorders. In particular, c.424C>T (p.Arg142Trp) is a documented *PRPH2* missense variant that was firstly identified in patients from the Netherlands [23]. However, its effect on the clinical phenotype remains controversial. Interestingly, some research studies described this variant as non-penetrant, meaning that individuals carrying it may remain asymptomatic for many years, with symptoms potentially appearing in later stages of life (76 years of age) [23]. On the other hand, other studies reported c.424C>T as a common causative variant in the European population associated with *PRPH2*-related retinal dystrophy with an autosomal dominant pattern [24]. In the present study, c.424C>T was identified in a 32-year-old Italian patient diagnosed with retinal dystrophy, highlighting a potential role in early-onset disease. The substitution may affect the protein structure and function due to the main biochemical differences between Arginine (Arg) and Trp. In particular, Arg is a basic and positively charged amino acid, playing an essential role in maintaining protein structural stability and facilitating functional interactions. In contrast, Trp is involved in protein folding and interacts with membranes. Moreover, the presence of Trp may cause conformational alterations that hamper the protein’s ability to package correctly into its functional conformation and interact properly with other molecules. As a result, c.424C>T may lead to substantial changes in the Prph2 protein, further supporting its involvement in the onset of IRDs. Of note, these findings emphasize how the same variant can lead to different phenotypic manifestations, confirming the existence of a wide spectrum of *PRPH2*-associated disorders. Another well-known pathogenic *PRPH2* variant identified in this cohort is c.458A>G (p.Lys153Arg). Although Lysine (Lys) and Arg share similar biochemical properties, small differences can influence protein conformation. This substitution may disrupt electrostatic interactions, leading to structural changes that could affect protein stability and folding. This variant was reported in patients with autosomal dominant *PRPH2*-associated retinal dystrophy [25,26] and was also identified in our cohort, in a patient with a family history of RP. However, the lack of instrumental data in patients with the c.424C>T and c.458A>G variants complicated the establishment of an accurate genotype–phenotype correlation. Regarding the c.493T>C (p.Cys165Arg) variant, it has been described in subjects with IRDs characterized by adult onset, good BCVA, and phenotypic heterogeneity [21]. In the present study, the c.493T>C variant was identified in a family with a diagnosis of Stargardt-like macular dystrophy with an age of onset of about 50 years. Ophthalmic examination of the proband revealed mild reduction in retinal thickness, excellent BCVA in both eyes, no significant alteration of retinal function under low-light conditions, improved retinal function under high light conditions, and a progressive loss of visual field sensitivity (Table 2 and Figure 3). These findings were consistent with the scientific literature, highlighting that central vision remains unaffected and retinal function is not substantially compromised in the presence of this variant. In terms of the effect of amino acid substitution, the replacement of Cysteine (Cys) with Arg prevents the creation of disulphide bonds, destabilizing and disrupting protein folding. This substitution also modifies Prph2 interactions with cell membranes in rods, potentially contributing to the deterioration of peripheral vision. These data support the role of the c.493T>C variant in the onset and progression of IRDs, although a precise genotype–phenotype correlation remains unclear. Similarly, another variant with a pathogenic effect is c.499G>A (p.Gly167Ser). It is involved in the onset of several IRDs such as Pattern Dystrophy, Extensive Chorioretinal Atrophy, Pattern Dystrophy simulating Fundus Flavimaculatus, and autosomal-dominant RP, highlighting the considerable phenotypic heterogeneity associated with *PRPH2* [22]. This substitution involves amino acid with consistent biochemical differences, affecting the global architecture of the Prph2 protein. In fact, replacing Glycine (Gly) with Ser modifies the protein’s secondary structure and reduces its flexibility. As reported in several scientific articles, this substitution occurs in the intradiscal loop (ID2) and affects the protein function [22,27]. In the present study, this variant was detected in a patient with a family history of visual impairment and clinical diagnosis of rod–cone dystrophy. This finding supports the existence of an autosomal dominant inheritance pattern, confirming the role of c.499G>A in the manifestation of *PRPH2*-associated disorders. However, the absence of available instrumental data impaired a precise genotype–phenotype correlation. Another variant with pathogenic effect is c.612C>G (p.Tyr204Ter), one of the most commonly reported truncating variants associated with *PRPH2*-related retinopathy [27,28,29]. This variant leads to LOF, generating a protein truncation or NMD. The variant was identified in a patient with a clinical diagnosis of cone–rod dystrophy and segregation analysis revealed that her affected daughter carries the same pathogenic variant, supporting an autosomal dominant inheritance pattern. In particular, the instrumental evaluations performed on the proband suggested a progressive retinal degeneration affecting both rod and cone function, which is consistent with the diagnosis. In addition, the preserved peak times in both scotopic and photopic ERGs suggest that the remaining retinal cells are not completely damaged (Table 2). On the other hand, ophthalmic examinations performed on the daughter of the proband showed increased retinal thickness in the left eye, which may indicate an active pathological process, such as edema or intraretinal accumulation. BCVA and CV FDT measurements reported that central vision was better in the right eye than in the left, consistent with the OCT results. Both eyes also displayed reduced scotopic and photopic amplitudes, pointing to the degeneration of rods and cones, which are typical signs of retinal dystrophies (Table 2). The disease progression appeared to be moderate and the central visual function was still relatively well-preserved. A clinical comparison between mother and daughter revealed different stages of the same inherited condition affecting both rods and cones. The mother showed a more advanced stage of disease, consistent with her age, while the daughter displayed early-stage alterations in line with the onset of disease and a slow disease progression with good preservation of central vision.

The known variant c.461_463del (p.Lys154del) has been reported to be associated with a range of retinal phenotypes, from RP to macular dystrophy [30]. Although this variant has a dominant effect on both cone and rod function, it may enhance rod structure while compromising cone structure. A research study suggested that c.461_463del may promote rod OS morphogenesis, although it may not necessarily preserve OS function. This condition may explain the clinical heterogeneity observed among patients with the same pathogenic variant [31]. In our cohort, this variant was identified in a single patient with RP and the instrumental data supported the clinical diagnosis. The CV FDT revealed a substantial rod dysfunction characterized by early peripheral vision loss, whereas OCT data may indicate ongoing degenerative changes suggestive of a slowly progressive disease course (Table 2). These findings allowed a precise genotype–phenotype correlation between the c.461_463del variant and RP. Furthermore, the c.734dup (p.Trp246ValfsTer55) variant was also described in association with different IRDs [32,33]. In particular, Antonelli G et al. reported that the effect of c.734dup on the phenotype may be modulated by different variants in the *ABCA4* gene [32]. In contrast to this finding, our patients displayed the c.734dup variant in the absence of other modifier genes, suggesting a specific causative role in IRDs, rather than acting as a modifier in combination with variants in other genes. This pathogenic variant was found in two unrelated subjects with a clinical suspicion of IRDs. In particular, the ocular evaluation of our patients revealed good visual acuity in both eyes and a reduction in central visual sensitivity, suggesting subclinical retinal changes (Table 2). These clinical data suggest the presence of a mild form of cone dystrophy or a hereditary macular dystrophy caused by the c.734dup variant, which may have a crucial role in the pathogenesis of IRDs. Longitudinal clinical assessment was available exclusively for pt-11A, as illustrated in Figure 3.

Overall, this study emphasizes the phenotypic heterogeneity associated with *PRPH2*, highlighting how intra- and inter-individual variability complicates a precise genotype–phenotype correlation. These findings suggest the presence of potential factors, which may influence the onset, severity, and progression of *PRPH2*-associated disorders. It is important to note that some patients lacked instrumental data such as BCVA, OCT, or ERG, which limits the confidence in genotype–phenotype correlations for those cases. Consequently, genotype–phenotype analyses were focused on available clinical and genetic data and supplemented with evidence from the literature when variants were previously reported. Given these premises, further investigations will be essential to identify possible phenotype modifiers and to elucidate the effect of *PRPH2* variants on protein structure and function, as well as on the onset and progression of IRDs. Moreover, the present study identified three novel *PRPH2* variants (c.653C>G, c.700T>C, and c.121del), expanding the spectrum of known pathogenic variants involved in the etiopathogenesis of IRDs and further highlighting the relevance of the WES approach to facilitate molecular diagnosis.

In conclusion, WES analysis revealed 10 different variants in the *PRPH2* gene in a cohort of 830 individuals with IRDs, confirming the rarity of *PRPH2*-associated disorders. These findings further highlight the wide range of clinical manifestations caused by *PRPH2* variants, emphasizing the importance of combining genetic, clinical, and instrumental data to improve our understanding of disease mechanisms. This approach enhances diagnostic accuracy, facilitates more precise genotype–phenotype correlations, and ultimately optimizes patient stratification and care. From a clinical perspective, early molecular diagnosis of *PRPH2*-related disease could help to differentiate it from conditions with similar phenotypes, such as age-related macular degeneration or Stargardt disease. Additionally, it could facilitate the realization of a multimodal platform designed to enhance decision-making strategies for supporting clinicians in the management of these conditions. This would facilitate more accurate counselling of patients, enable genetic testing for family members, and potentially allow timely inclusion in gene-targeted therapeutic trials. Although no therapies are currently approved for *PRPH2*-associated IRDs and no treatments are under active development, this research study offers encouraging prospects. Further studies, including natural history data and functional validation of variants, are essential to support the development of effective therapies and their integration into routine clinical management.

## Figures and Tables

**Table 1:** Variant characterization and classification.

Gene	Chromosome Position	Variant Nomenclature	Variant Type	GnomAD Frequency	Variant Effect on Protein	Protein Domain (Localization)	ACMG Classification
*PRPH2* (NM_000322.5)	6:42672278	c.653C>G (p.Ser218Trp)	Missense	-	Structural damage detected	Topological (ID)	Pathogenic
	6:42672231	c.700T>C (p.Tyr234His)	Missense	-	Debated	Topological (ID)	VUS
	6:42689952	c.121del (p.Leu41Ter)	Nonsense	-	Creates a premature stop codon	Transmembrane (Disc membrane)	Pathogenic
	6:42689649	c.424C>T (p.Arg142Trp)	Missense	0.00000879	Affects function	Topological (ID)	Pathogenic
	6:42689615	c.458A>G (p.Lys153Arg)	Missense	-	Affects function	Topological (ID)	Pathogenic
	6:42689610	c.461_463del (p.Lys154del)	Deletion	-	Probably affects function	Topological (ID)	Pathogenic
	6: 42689580	c.493T>C (p.Cys165Arg)	Missense	-	Affects function	Topological (ID)	Pathogenic
	6:42689574	c.499G>A (p.Gly167Ser)	Missense		Affects function	Topological (ID)	Pathogenic
	6:42672319	c.612C>G (p.Tyr204Ter)	Nonsense	-	Creates a premature stop codon	Topological (ID)	Pathogenic
	6:42672197	c.734dup (p.Trp246ValfsTer55)	Insertion	-	Probably affects function	Topological (ID)	Pathogenic

**Table 2:** Summary of ophthalmic and genetic findings.

Patient ID	Clinical Diagnosis	Main Visual Symptoms	Variant	Inheritance Pattern	Notable Changes
**pt-1A**	macular dystrophy	reduced acuity, photophopia	c.653C>G (p.Ser218Trp)	-	reduced scotopic and photopic ERGs
**pt-2A**	cone–rod dystrophy	reduced acuity, photophopia, nictalopia	c.700T>C (p.Tyr234His)	AD	reduced scotopic and photopic ERGs
**pt-6A**	Stargardt-like macular dystrophy	reduced acuity, photophopia	c.493T>C (p.Cys165Arg)	AD	reduced scotopic and photopic ERGs
**pt-8A**	cone–rod dystrophy	reduced acuity, photophopia	c.612C>G (p.Tyr204Ter)	AD	altered dark adaptation
**pt-8B**	cone–rod dystrophy	reduced acuity, photophopia	c.612C>G (p.Tyr204Ter)	AD	altered dark adaptation
**pt-9A**	RP	reduced acuity, photophopia	c.461_463del (p.Lys154del)	-	altered dark adaptation
**pt-10A**	cone–rod dystrophy	reduced acuity, photophopia	c.734dup (p.Trp246ValfsTer55)	AD	altered dark adaptation
**pt-11A**	macular dystrophy	reduced acuity, photophopia	c.734dup (p.Trp246ValfsTer55)	-	altered dark adaptation

## Data Availability

The original contributions presented in this study are included in the article/Supplementary Material. Further inquiries can be directed to the corresponding author.

## Supplementary Materials

The following supporting information can be downloaded at: https://www.mdpi.com/article/10.3390/biomedicines13071531/s1. Figure S1: Representative examples of three identified missense variants, including sequencing read alignments and Sanger validation; Table S1: List of analyzed genes; Table S2: Instrumental data collected from patients harbouring *PRPH2* variants; na indicates data not available.

## Abbreviations

The following abbreviations are used in this manuscript:

RP	Retinitis Pigmentosa
BVCA	Best Corrected Visual Acuity
OCT	Optical Coherence Tomography
ERG	Electroretinography
CV FDT MD	Frequency Doubling Technology Visual Field Testing
SNVs	Single Nucleotide Variants
InDels	Insertions and Deletions
MAF	Minor Allele Frequency
ACMG	American College of Medical Genetics and Genomics
ACGS	Association for Clinical Genomic Science
VUS	Variant with Uncertain Significance
REVEL	Rare Exome Variant Ensemble Learner
