# Supplementary material for: Expanding Genetic and Clinical Spectra of Inherited Retinal Dystrophies: Identification of Three Novel PRPH2 Variants
Source: Biomedicines. 2025 Jun 23;13(7):1531. doi: 10.3390/biomedicines13071531 (PMC12292585; doi:10.3390/biomedicines13071531)
Supplement: Supplementary file 1 [file biomedicines-13-01531-s001.zip › supplementary table 2.pdf]

Table S2. Instrumental data collected from patients harboring PRPH2 variants, na indicates data not available

Patient ID	OD									OS							
	Date Exam	BCVA		OCT CRT	ERG				CV FDT MD	BCVA		OCT CRT	ERG				CV FDT MD
		Log MAR	Letter		Scoto Pic	Peak Time	Photo pic	Peak Time		Log MAR	Lette r		Scoto pic	Peak Time	Photo pic	Peak Time	
**pt-1A**	Jan. 2022	na	na	na	39,36	48,63	17,02	32,81	na	na	na	na	37,6	49,22	20,08	32,81	na
**pt-2A**	Oct. 2018	0.6	55	174	29,04	53,32	13,88	37,89	na	1.0	34	164	29,51	55,08	9,98	40,23	na
	Oct. 2019	0.9	39		39,82	58,01	16,45	36,72	-8,16	1.0	34		41,53	59,18	13,84	37,89	-11,59
**pt-6A**	Dec. 2021	0.0	84	267	21,78	57,42	8,29	38,67	-14,57	0.0	84	247	17,6	56,84	6	37,5	-14,57
	Dec. 2022	0.0	84	251	22,62	56,84	13,59	37,89	-18,24	0.0	84	238	26,52	57,42	11,62	37,5	-19,75
**pt-8A**	Sept. 2020	0.3	70	na	39,73	56,25	24,89	37,5	-14,69	0.5	59	na	37,15	58,59	19,83	37,89	-15,66
**pt-8B**	Oct. 2020	0.1	82	278	40,68	53,91	13,65	32,81	-5,99	0.2	75	329	44,5	55,8	13,65	32,81	-4,08
**pt-9A**	Dec. 2017	na	na	270	na	0,27	na	na	-27,46	na	na	271	na	na	na	na	-25,27
	Jan. 2019	na	na	na	na	na	na	na	na	na	na	266	na	na	na	na	-24,37
	Mar. 2019	na	na	251	na	na	na	na	na	na	na	263	na	na	na	na	Na
	Sept. 2019	na	na	269	na	na	na	na	-27,43	na	na	264	na	na	na	na	-24,87
	July 2020	na	na	263	na	na	na	na	-26,97	Na	na	273	na	na	na	na	-24,97
	Aug. 2020	na	na	308	na	na	na	na	na	Na	na	285	na	na	na	na	na
	Dec. 2020	na	na	252	na	na	na	na	na	Na	na	291	na	na	na	na	na
	Nov. 2021	na	na	278	na	na	na	na	na	Na	na	293	na	na	na	na	na
**pt-10A**	Mar. 2022	0.1	83	na	na	na	na	na	-19,1	0.0	85	na	na	na	na	na	-16,1
**pt-11A**	Feb. 2020	na	na	257	56,56	50,39	9,32	33,98	-1,73	Na	na	259	17,89	52,73	9,75	33,59	-2,39
	Mar. 2021	na	na	257	23,7	56,84	15,9	34,38	-1,83	Na	na	281	18,89	56,25	13,76	33,98	-3,96
	Feb. 2023	0.2	77	252	23,33	49,8	9,74	34,38	-1,15	0.1	79	266	16,35	50,98	8,35	35,42	-2,28
