# Supplementary figures and images for: Expanding Genetic and Clinical Spectra of Inherited Retinal Dystrophies: Identification of Three Novel PRPH2 Variants
Source: Biomedicines. 2025 Jun 23;13(7):1531. doi: 10.3390/biomedicines13071531 (PMC12292585; doi:10.3390/biomedicines13071531)

## Slide 1
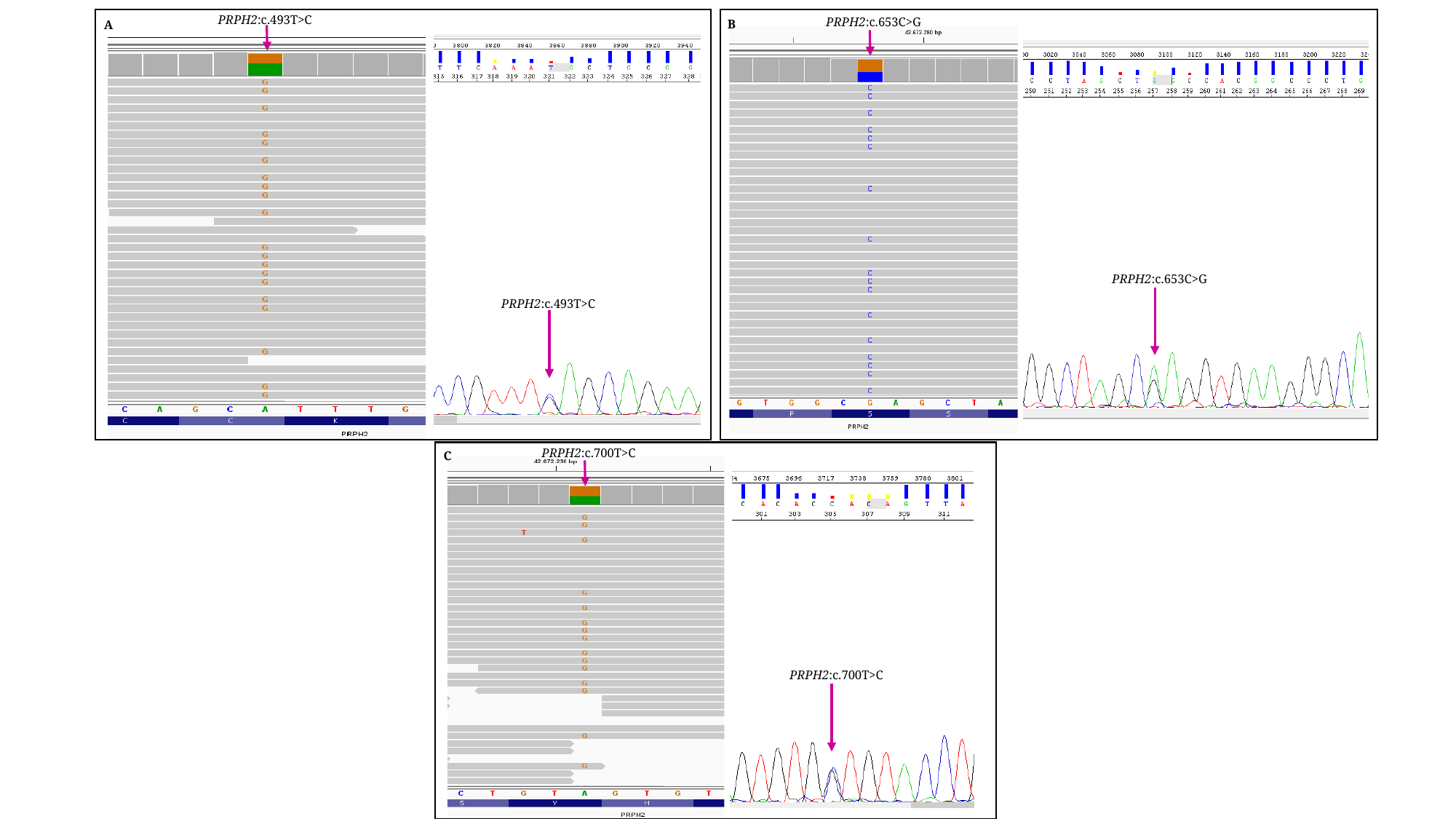

PRPH2:c.493T>C
PRPH2:c.493T>C
PRPH2:c.653C>G
PRPH2:c.653C>G
B
A
PRPH2:c.700T>C
PRPH2:c.700T>C
C

Supplement: Supplementary file 1 [file biomedicines-13-01531-s001.zip › Figure S1.pptx]
